# Comparative transcriptomic analysis of long noncoding RNAs in *Leishmania*-infected human macrophages

**DOI:** 10.3389/fgene.2022.1051568

**Published:** 2023-01-04

**Authors:** Juliane C. R. Fernandes, André N. A. Gonçalves, Lucile M. Floeter-Winter, Helder I. Nakaya, Sandra M. Muxel

**Affiliations:** ^1^ Departamento de Fisiologia, Instituto de Biociências, Universidade de São Paulo, São Paulo, Brazil; ^2^ Instituto de Medicina Tropical da Faculdade de Medicina da Universidade de São Paulo, São Paulo, Brazil; ^3^ Scientific Platform Pasteur-USP (SPPU), São Paulo, Brazil; ^4^ Hospital Israelita Albert Einstein, São Paulo, Brazil; ^5^ Instituto de Ciências Biomédicas, Universidade de São Paulo, São Paulo, Brazil

**Keywords:** gene expression, host-parasite interaction, lncRNA, THP-1, RNA-seq, transcriptomics, *Leishmania*, macrophage

## Abstract

It is well established that infection with *Leishmania* alters the host cell’s transcriptome. Since mammalian cells have multiple mechanisms to control gene expression, different molecules, such as noncoding RNAs, can be involved in this process. MicroRNAs have been extensively studied upon *Leishmania* infection, but whether long noncoding RNAs (lncRNAs) are also altered in macrophages is still unexplored. We performed RNA-seq from THP-1-derived macrophages infected with *Leishmania amazonensis* (*La*), *L. braziliensis* (*Lb*)*,* and *L. infantum* (*Li*)*,* investigating a previously unappreciated fraction of macrophage transcriptome. We found that more than 24% of the total annotated transcripts and 30% of differentially expressed (DE) RNAs in *Leishmania*-infected macrophage correspond to lncRNAs. LncRNAs and protein coding RNAs with altered expression are similar among macrophages infected with the *Leishmania* species. Still, some species-specific alterations could occur due to distinct pathophysiology in which *Li* infection led to a more significant number of exclusively DE RNAs. The most represented classes among DE lncRNAs were intergenic and antisense lncRNAs. We also found enrichment for immune response-related pathways in the DE protein coding RNAs, as well as putative targets of the lncRNAs. We performed a coexpression analysis to explore potential cis regulation of coding and antisense noncoding transcripts. We identified that antisense lncRNAs are similarly regulated as its neighbor protein coding genes, such as the BAALC/BAALC-AS1, BAALC/BAALC-AS2, HIF1A/HIF1A-AS1, HIF1A/HIF1A-AS3 and IRF1/IRF1-AS1 pairs, which can occur as a species-specific modulation. These findings are a novelty in the field because, to date, no study has focused on analyzing lncRNAs in *Leishmania*-infected macrophage. Our results suggest that lncRNAs may account for a novel mechanism by which *Leishmania* can control macrophage function. Further research must validate putative lncRNA targets and provide additional prospects in lncRNA function during *Leishmania* infection.

## 1 Introduction

Endogenous noncoding (nc) regulatory RNAs are classified according to their length: the small noncoding RNAs (sncRNAs), of which the 25 nucleotides-long microRNAs (miRNAs) exert regulatory functions, and the long noncoding RNAs (lncRNAs), which are larger than 200 nucleotides. LncRNAs were classified based on biogenesis signatures, such as genomic position and proximity to protein coding genes: as sense intergenic (IG), overlapping (OT), and intronic (IT), RNAs from the same orientation of closely related protein-coding genes in their genome *loci*, and antisense (AS) RNAs, transcribed from the opposite strand of protein coding genes (Fernandes et al., 2019). LncRNAs are transcribed by RNA polymerase II but differ from mRNAs since the transcripts can assume multiple forms. The linear transcripts can be polyadenylated or not; alternatively, lncRNA can be circularized, forming circular (circ) RNAs (Zhang et al., 2013).

Unlike miRNAs that mostly perform posttranscriptional modulation of target genes through 3′UTR recognition, lncRNAs act by transcriptional to posttranslational mechanisms, regulating many physiological and pathological processes (Fernandes et al., 2019). LncRNAs can regulate their molecular targets by multiple mechanisms at both the nucleus and the cytoplasm, such as regulating neighbor genes (Joung et al., 2017), mediating protein function through direct structural association (Rinn & Chang, 2012), and even by sponging miRNAs (Ebert et al., 2007).

Also, lncRNAs are strong components in defining cell phenotype and function. The transcriptome of T lymphocytes during development and differentiation showed that more than 50% of the identified lncRNAs are stage-specific. At the same time, the coding transcripts fraction is mainly shared among the compared groups (Hu et al., 2013). Similarly, lncRNA signature in primary or THP-1-derived human macrophages stimulated either with IFN-γ plus LPS or IL-4 induced a subset of lncRNAs defining macrophage phenotype, and RNA interference (RNAi) of specific lncRNAs prevented the expression of macrophage polarization markers (Huang et al., 2016).

LncRNAs are mediators of immune response (Chen et al., 2017; de Lima et al., 2019) and act at both pro- and anti-inflammatory (Du et al., 2017) pathways in macrophages. There is an increasing interest in ncRNA biology and function in the context of host-parasite interaction, but most studies focus on miRNAs (Bayer-Santos et al., 2017; Bensaoud et al., 2019). Pathogen-mediated change in lncRNAs expression has been studied in macrophages infected with the parasitic protozoa *Toxoplasma gondii* (Menard et al., 2018), fungal infection by *Cryptococcus neoformans* (Gao et al., 2022), and in bacterial infections, such as *Mycobacterium tuberculosis* (Yang et al., 2016) and *Salmonella typhimurium* (Westermann et al., 2016).

Twenty different species of the *Leishmania* genus cause 0.7 to 1 million new human cases of leishmaniasis each year worldwide (Burza et al., 2018). The disease can cause ulcers in the skin or mucosa that can be self-healing or cause organ damage, mainly in the liver, spleen, and bone marrow, within three main clinical forms: cutaneous, mucocutaneous, and visceral leishmaniasis.

After the transmission of *Leishmania* promastigote forms by infected sandflies, the parasites differentiate to amastigotes inside phagocytic cells in the mammalian host. *Leishmania* establishes its replicative niche primarily in the phagolysosome of macrophages. Disease outcome reflects the balance between pro-inflammatory macrophages with M1 phenotype and anti-inflammatory M2 macrophages induced by *Leishmania*’s immune response subversion mechanisms (Soong, 2012). Multiple biological processes are involved in the macrophage response, such as the metabolism (Muxel et al., 2018; Ferreira et al., 2021) and cytokine production (Zamboni & Sacks, 2019).

Previous studies determined the transcriptome of *Li* infection in THP-1 derived macrophages (Gatto et al., 2020), *La* and *L. major* infection in primary human macrophages (hMDM) (Fernandes et al., 2016) and *Lb* infection in patient’s lesions (Maretti-Mira et al., 2012). In murine macrophages, the transcriptome of BALB/c and C57BL/6 macrophages infected with *La* indicated an inflammatory response different from the spectrum extremes M1 and M2 polarized macrophages (Osorio y Fortéa et al., 2009; Aoki et al., 2019) observed in *L. major* (Sacks & Noben-Trauth, 2002). All of the above-mentioned results of transcriptome-wide experiments exhibit some contrasting results with the literature because macrophage response to *Leishmania* is highly dependent on the parasite species and strain and host cell type, thus the importance of investigating different models (Salloum et al., 2021). The transcriptome of macrophages is widely affected by inflammatory stimuli (Das et al., 2018; Vollmers et al., 2021). Changes in gene expression upon *Leishmania* infection have been documented in a variety of *Leishmania*-host cell models, explaining different aspects of immune response subversion elicited by this parasite (Salloum et al., 2021). But, to our knowledge, no data is available that systematically compares the transcriptomic profile of macrophages infected with *La*, *Lb,* or *Li*.

Besides these transcriptomic studies on genes that encode proteins, ncRNAs in *Leishmania* infection of both murine and human macrophages were also investigated, focusing primarily on miRNAs and their role in regulating mRNAs related to the inflammatory response (Lemaire et al., 2013; Geraci et al., 2015; Muxel et al., 2017; Colineau et al., 2018; Diotallevi et al., 2018; Kumar et al., 2020; Souza et al., 2021; Ramos-Sanchez et al., 2022). Some groups dedicate to identifying and characterizing *Leishmania*’s subsets of ncRNAs regulating parasite developmental stages, and although we did not evaluated parasite’s reads, these groups can benefit from our publicly available datasets (Dumas et al., 2006; Freitas Castro et al., 2017; Ruy et al., 2019).

LncRNAs were not investigated in *Leishmania* infection until recently. Sanz and collaborators have identified 21 differentially expressed lncRNA in the lymph nodes of dogs infected with *L. infantum* (Sanz et al., 2022). Maruyama and collaborators explored lncRNA content in the blood transcriptome of visceral leishmaniasis patients infected with *L. infantum* (Maruyama et al., 2022). Still, how different species of *Leishmania* parasites affect macrophage transcriptome is an open question. In this study, we investigated human macrophages infected with *Leishmania amazonensis* (*La*), *L. braziliensis* (*Lb*), and *L. infantum* (*Li*), the causative agent of cutaneous, mucocutaneous and visceral leishmaniasis, respectively (Burza et al., 2018).

Here we show the coding and noncoding RNA profile of THP-1-derived macrophages infected with *La*, *Lb*, or *Li*. We focused on describing dysregulated lncRNAs, comparing their specificity upon infection by different *Leishmania* species, pathway enrichment analysis based on mRNA profile and putative lncRNA targets, lncRNA-mRNA pairs with close genomic *loci* coexpression, and highlighting prospects on the study of macrophage ncRNA function in *Leishmania* infection.

## 2 Results

### 2.1 Modulation of protein coding and long noncoding host RNAs upon *Leishmania* infection

To compare transcripts involved in the infection by *La*, *Lb,* and *Li*, we performed RNA-seq of human THP-1-derived macrophages infected with these species for 24 h (Supplementary Figure S1); in this time, infection is established, and the transcriptomic alterations related to lncRNAs would be relevant to study in the early phase of infection and their implications in the activation to immune response. The high-quality reads were deposited at NCBI under BioProject with the accession number PRJNA881925. We identified an average of 41 million reads per sample (with an average of 40% reads mapped for the human genome for infected macrophage samples and 99% for uninfected macrophages), resulting in 19043 genes mapped to the GRCh38 human genome. From that, 4,311 were differentially expressed (DE) in at least one infection model compared to uninfected macrophages. The most abundant transcripts were protein coding and long noncoding RNAs (lncRNAs) (Table 1).

**TABLE 1:** Number and percentage of the different classes of annotated transcripts. Columns depict the number (#) of transcripts either from total mapped transcripts or from differentially expressed (DE) from all *Leishmania*-infected macrophages and the percentage (%) for each category. LncRNA, long noncoding; miRNA, microRNA; snoRNA, small nucleolar RNA; snRNA, small nuclear RNA; rRNA, ribosomal RNA.

Category	# from total transcripts	% from total	# from DE	% total DEGs
Protein coding	13892	72.95	2,926	67.87
LncRNA	4,627	24.30	1,267	29.39
miRNA	139	0.73	29	0.67
snoRNA	121	0.64	22	0.51
snRNA	33	0.17	20	0.46
rRNA	4	0.02	4	0.09
Others	227	1.19	43	1
Total	19043		4,311	

All infected samples presented similar scores for molecular degree of perturbation (MDP, Supplementary Figure S2A), and no outlier was found in our samples (Gonçalves et al., 2019). We performed principal component analysis (PCA, Supplementary Figure S2B), showing that 50% of the total variance is explained by the differences among uninfected and *Leishmania*-infected macrophages in dimension 1 (Dim1). In comparison, Dim2 further explains 14% of the variance by clustering *La* and *Lb*-infected macrophage samples away from *Li*-infected macrophage samples.

Since lncRNAs are important regulators of mRNA expression and those were the two most abundant transcripts identified as DE, we investigated the number of DE protein coding and lncRNA transcripts identified in the RNA-seq of THP-1 macrophages infected with *La*, *Lb* or *Li* compared to uninfected macrophages (Supplementary Table S1). We identified a total of 1503 DE protein coding transcripts (883 up- and 620 downregulated) in *La*-infected macrophages, 1708 DEs (984 up- and 724 downregulated) in *Lb*-infected macrophages, and 2,572 DEs (1,285 up- and 1,287 downregulated) in *Li*-infected macrophages (Supplementary Table S1). From the lncRNA-annotated subset, we identified a total of 735 DE lncRNAs (17% of DE, 335 up- and 400 downregulated) for *La*-infected macrophages, 795 DE lncRNAs (13% of DE, 380 up- and 415 downregulated) for *Lb*-infected macrophages and 1082 DE lncRNAs (11% of DE, 466 up- and 616 downregulated) for *Li*-infected macrophages (Supplementary Table S1).

**FIGURE 1:**
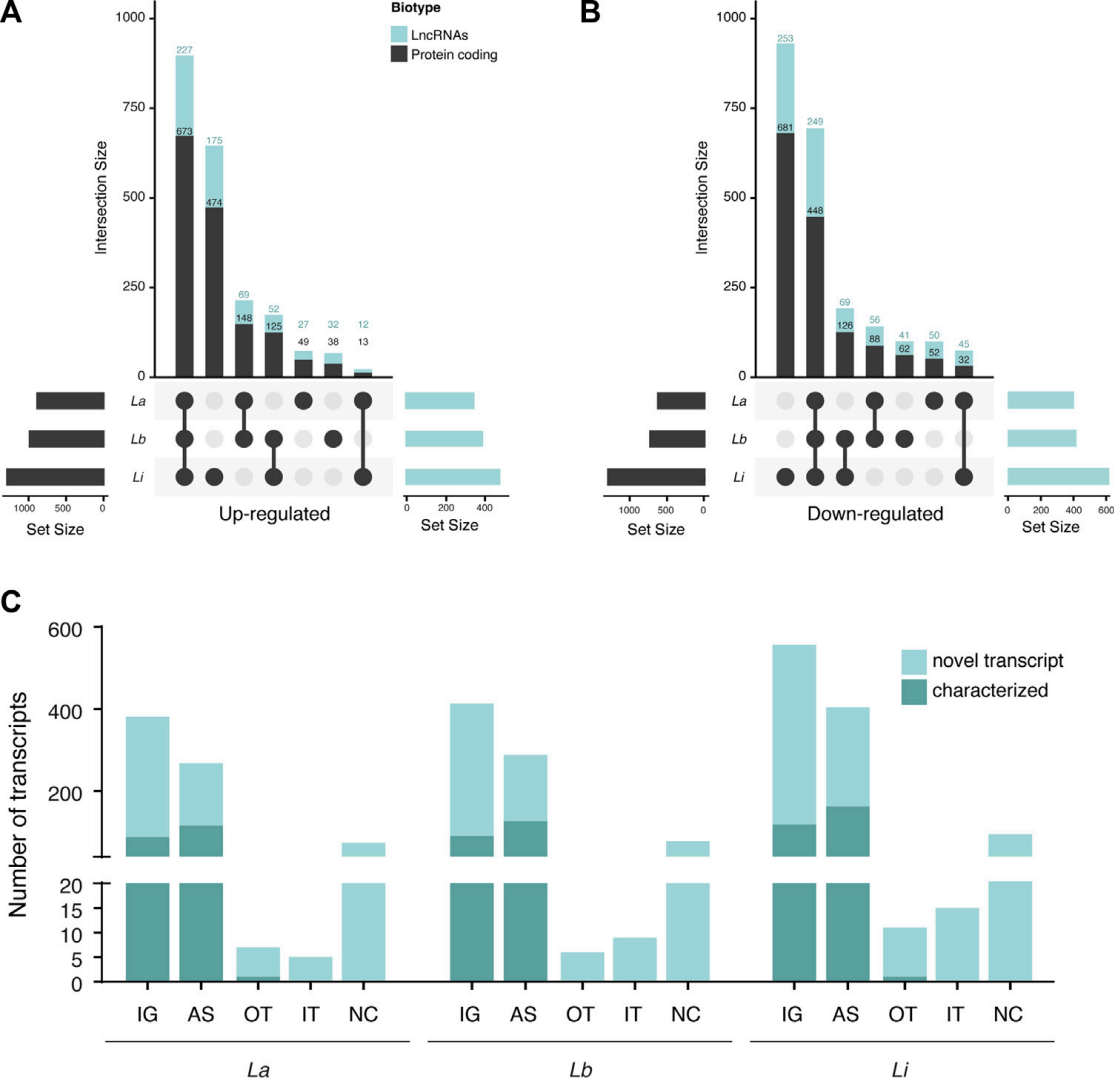
Number of differentially expressed protein coding and long noncoding RNAs subtypes in *Leishmania*-infected THP-1 macrophages. **(A)** Intersection plot representing the number of significantly upregulated lncRNAs and protein coding genes from *La*-, *Lb*- and *Li*-infected THP-1 macrophages (*N* = 5); **(B)** Intersection plot representing the number of significantly downregulated lncRNAs and protein coding genes from *La*-, *Lb*- and *Li*-infected THP-1 macrophages (*N* = 5); **(C)** Number of transcripts of each lncRNA type classified using the GRCh38.p13 database. The transcripts from the GRCh38.p13 database that lack validation studies are referred to as novel transcripts. IG: intergenic, AS: antisense; OT: overlapping transcript; IT: intronic transcript; NC: non-classified.

To observe the similarities of modulated protein coding transcripts (mRNAs) and lncRNAs during infection by different *Leishmania* species, in the intersection plot (Figures 1A,B), we compared the mRNAs and lncRNAs subsets in each model. Among the three infection models, we identified 673 commonly upregulated protein-coding genes (Figure 1A) and 448 commonly downregulated (Figure 1B). Also, 227 lncRNAs were upregulated regardless of parasite species (Figure 1A), and 249 had their expression reduced compared to uninfected macrophages (Figure 1B). The number of DEs specific for macrophages infected with one species vary. Most exclusively DE protein coding and lncRNAs appeared in *Li* infection, with 474 unique protein-coding and 175 lncRNAs up- and 681 unique protein-coding and 253 lncRNAs downregulated (Figures 1A,B). On the other hand, *La*-infected macrophage induced only 49 unique protein-coding and 27 lncRNAs up- and 52 mRNAs and 50 lncRNAs exclusively downregulated. For *Lb* infection, 38 mRNAs and 32 lncRNAs were specifically upregulated and 62 mRNAs and 41 lncRNAs were downregulated only in the infection by this species (Figures 1A,B).

We observed that the majority of DE lncRNAs were intergenic (IG) and antisense (AS) lncRNAs for *La*-, *Lb*- and *Li*-infected macrophages, part of them were classified as novel transcript (Figure 1C). Many transcripts remain unannotated and herein were included as non-classified (Figure 1C).

Our data showed a species-specific alteration in lncRNA and protein coding RNAs, in which DE transcripts from *Li* infection cluster away from *La* and *Lb* infection (Supplementary Figure S2). Also, *Leishmania* infection can regulate sense- (mainly intergenic) and antisense-encoded lncRNAs transcript in macrophages (Figure 1C).

Although not classified as a different class, as they can be intronic or intergenic, we have identified modulation of some microRNA host genes (miR-HGs). We saw the upregulation of the mature microRNA (miR)-155 and its precursor MIR155HG, which can function as lncRNA (Supplementary Table S1). We also identified other miR-HGs that act as lncRNAs, such as the upregulated MIR34AHG, MIR210HG, MIR9-1HG, MIR22HG, and MIR4713HG by all *Leishmania* species and the exclusively upregulated in *Li* infection, MIR23AHG. On the other hand, we saw downregulation of the MIR124-1HG, MIR4435-2HG, MIRLET7BHG, MIR3936HG, MIR222HG, MIR223HG and MIR9-3HG in all infection models and only found MIR181A2HG in *Li* infection (Supplementary Table S1).

### 2.2 Enrichment of immune response pathways-related gene sets upon *Leishmania* infection

**FIGURE 2:**
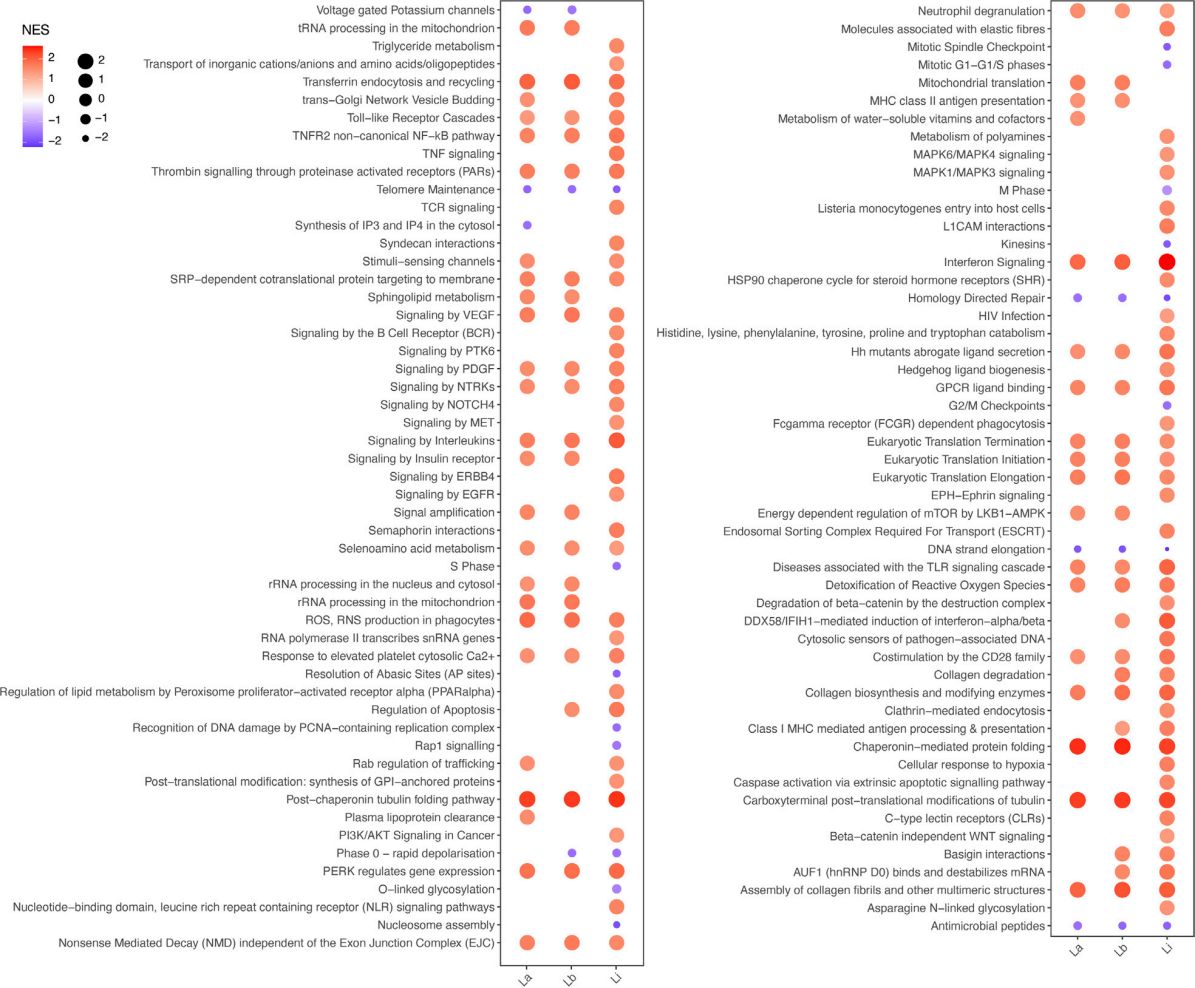
Enriched Reactome pathways for *La*, *Lb*, and *Li* infection in THP-1 macrophages. The plot represents the normalized enrichment score (NES) by both color and bubble size, as indicated in the figure legend, of significantly altered level 3 Reactome pathways based in the protein coding genes of THP-1 macrophages infected with *La*, *Lb* or *Li*.

We ran an enrichment analysis to investigate over-represented pathways within the identified DE protein coding genes. Level 3 Reactome pathways were ranked by the normalized enrichment score (NES) obtained through gene set enrichment analysis (GSEA) for THP-1 macrophages infected with *La*, *Lb,* and *Li* (Figure 2, Supplementary Table S2). We found that most of the significantly enriched immune response pathways in *Li*-infected macrophages are involved in parasite recognition, such as Toll-like receptor cascades, NLR signaling pathway, cytosolic sensors of pathogen-associated DNA, and C-type lectin receptor. In contrast, MHC class II antigen presentation was enriched for *La* and *Lb* infection.

**FIGURE 3:**
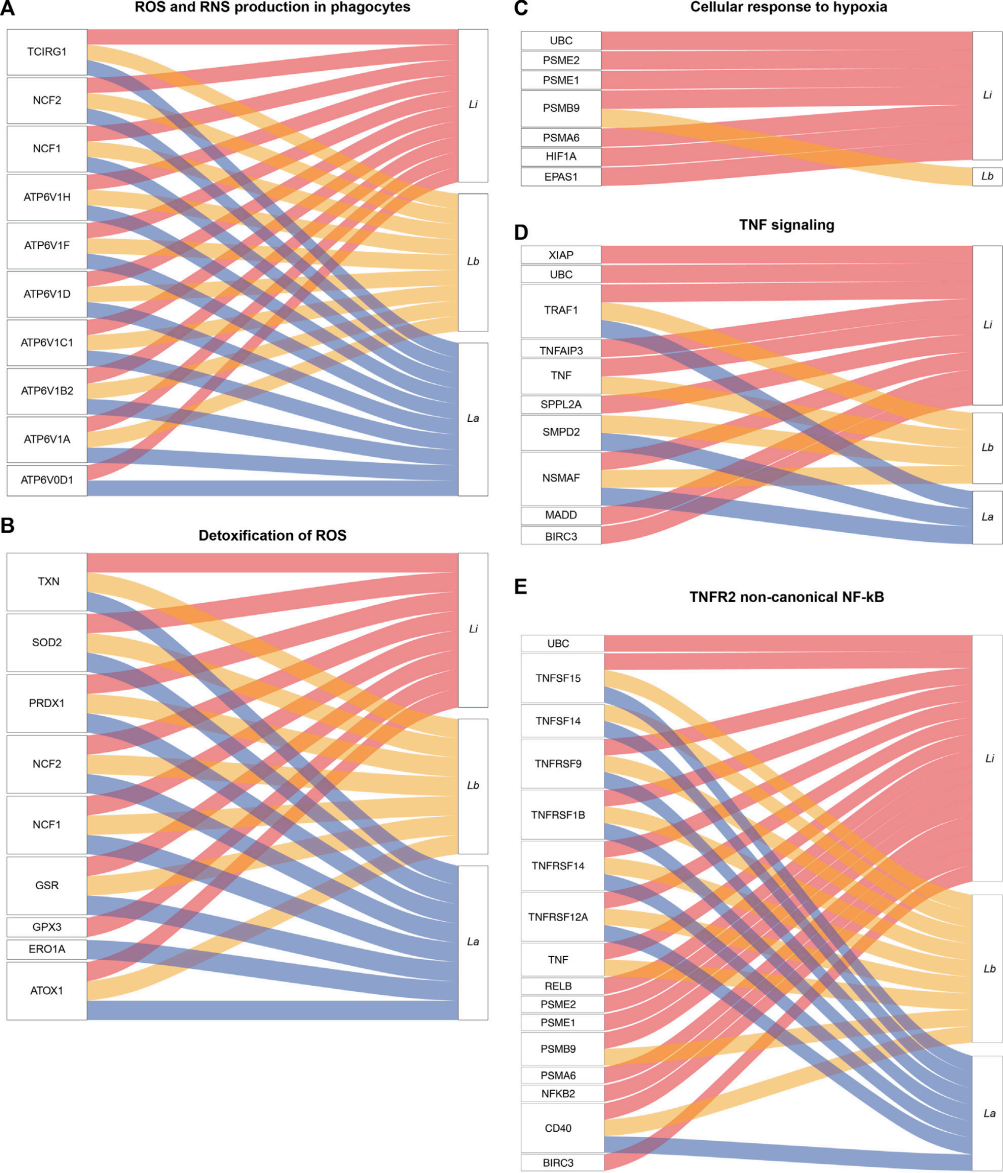
Alluvial plots representing enriched pathways and their related protein coding genes modulated in Leishmania-infected THP-1 macrophages. The alluvial plots show the significantly differentially expressed mRNAs in *La* (blue lines), *Lb* (yellow lines) and *Li* (red lines)-infected macrophages for 5 enriched pathways **(A)** Reactive oxygen species (ROS) and reactive nitrogen species (RNS) production in phagocytes **(B)** Detoxification of ROS **(C)** Cellular response to hypoxia **(D)** Tumor necrosis factor (TNF) signaling and **(E)** TNF receptor 2 (TNFR2) non-canonical nuclear factor kappa B (NF-κB).

Indeed, we extended our analysis to further evaluate species-specific activation of immune pathways, as shown by the cellular response to stimuli pathways. The pathway’s related DE genes, to a higher or lesser extent, are depicted in the alluvial plots (Figure 3). We found DE mRNAs related to reactive oxygen species (ROS) and reactive nitrogen species (RNS) production in phagocytes, such as Neutrophil NADPH Oxidase Factor 1 and 2 (NCF1 or p47phox and NCF2 or p67phox) and ATPase H + Transporting V1 Subunits B2, C1, D, F, H (ATP6V1), and detoxification of ROS, as thioredoxin (TXN), superoxide dismutase 2 (SOD2), Glutathione-Disulfide Reductase (GSR), Glutathione Peroxidase 3 (GPX3), and Peroxiredoxin 1 (PRDX1), in infection by all the three *Leishmania* species (Figures 3A,B, Supplementary Table S2).

We found DE mRNAs exclusively in *Li*-infected macrophages in pathways related to the cellular response to hypoxia, such as Ubiquitin C (UBC), genes of proteasome activation complex (PSME1, PSME2, PSMA6), Hypoxia Inducible Factor 1 Subunit Alpha (HIF1A), and Endothelial PAS Domain-Containing Protein 1 (EPAS1/HIF2A) (Figure 3C).

Among the enriched signal transduction pathways, we highlighted TNF signaling genes, such as X-Linked Inhibitor of Apoptosis (XIAP), an inhibitor of apoptosis Baculoviral IAP Repeat Containing 3(BIRC3), MAP Kinase Activating Death Domain (MADD), and UBC exclusively DE mRNAs in *Li*-infected macrophages, and DE mRNAs shared by all the three *Leishmania* species infection, as TNF, TNF Receptor Associated Factor 1 (TRAF1) and TNF Alpha Induced Protein 3 (TNFAIP3) (Figure 3D).

In the cytokine response mediated by tumor necrosis factor receptor 2 (TNFR2) non-canonical nuclear factor kappa B (NFκB) pathway, we found DE mRNAs exclusively in *Li*-infected macrophages, such as NFKB2 and RelB (Figure 3E) but also DE mRNAs shared by all the three *Leishmania* species infection, such as TNF Superfamily Member 14 and 15 (TNFSF14/15) and TNF receptor Superfamily Member 1B, 9, 12A and 14 (TNFRSF).

### 2.3 Antisense lncRNAs are coexpressed with sense protein coding genes during *Leishmania* infection and may be involved in immune response

**FIGURE 4:**
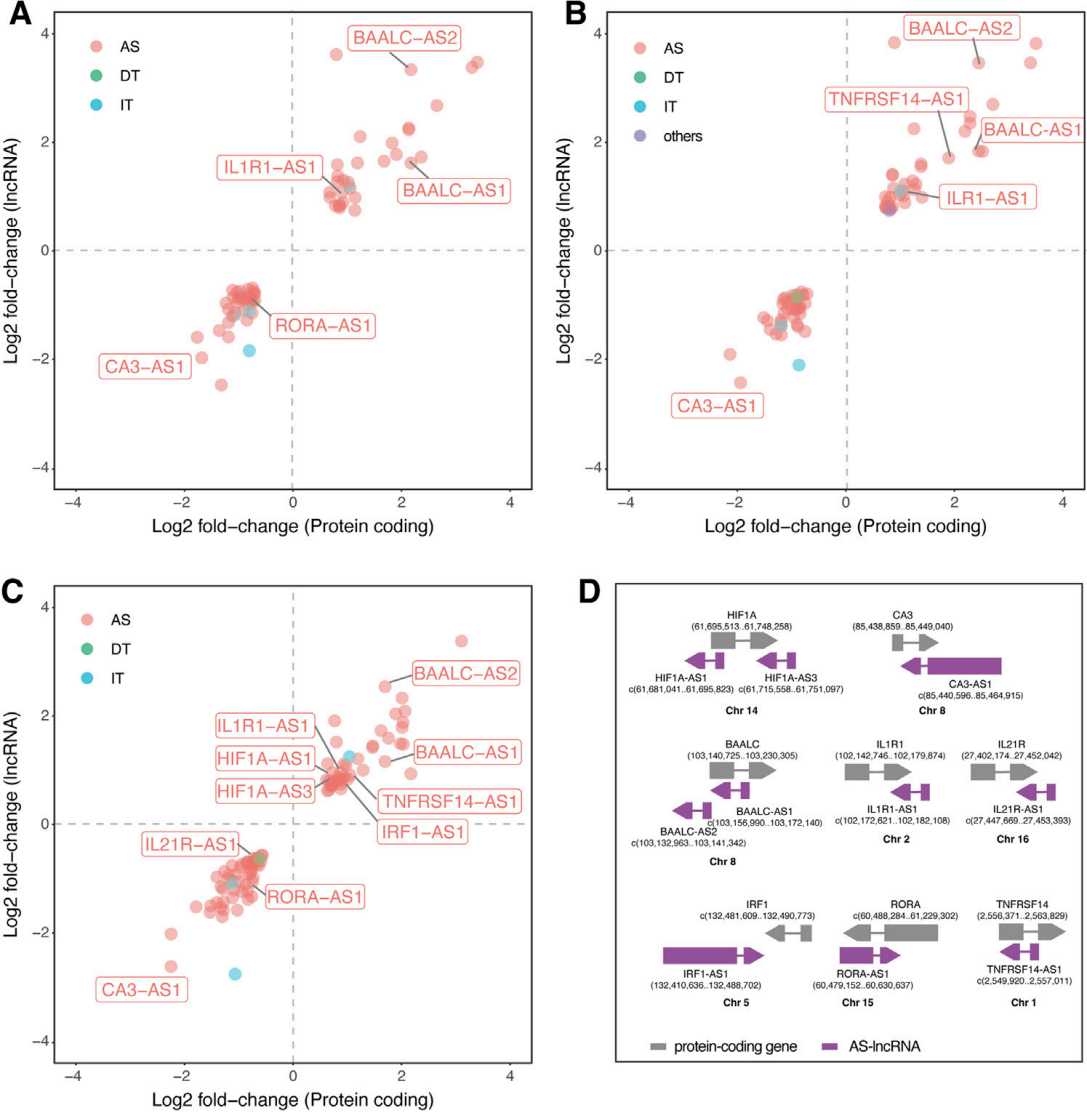
Coexpression of LncRNAs and mRNAs modulated upon *Leishmania* infection in THP-1 macrophages. Scatter plots represent the log2 fold-change values determined by RNA-seq of protein coding (X axis) and lncRNAs (Y axis) of **(A)** *L. amazonensis*-infected **(B)** *L. braziliensis*-infected **(C)** *L. infantum*-infected macrophages; **(D)** illustrations representing the genomic position of 10 coregulated lncRNA-mRNA pairs, in which grey represents protein-coding gene, purple represent AS-lncRNA and the numbers depict genomic position in the sense and antisense complementary DNA strand. AS: antisense; DT: divergent transcript; IT: intronic transcript; Chr: chromosome.

Since antisense lncRNAs correspond to a major regulated class during *Leishmania* infection, we ran a coexpression analysis to decipher whether the DE lncRNAs and their respective neighbor protein coding gene are simultaneously regulated and may be functionally connected through cis regulation. We found 69 lncRNA-mRNA pairs coregulated in *La*-infected macrophages, 77 in *Lb*-infected macrophages, and 101 in *Li*-infected macrophages (Figures 4A–C). Of those, we highlighted 10 antisense lncRNAs pairing to 8 genes previously described in *Leishmania*-infected macrophages with their genomic positions (Figure 4D): BAALC/BAALC-AS1, BAALC/BAALC-AS2, HIF1A/HIF1A-AS1, HIF1A/HIF1A-AS3, TNFRSF14/TNFRSF14-AS1, IRF1/IRF1-AS1, CA3/CA3-AS1, IL1R1/IL1R1-AS1, IL21R/IL21R-AS1, and RORA/RORA-AS1.

Correlation analysis showed a positive correlation between protein coding and AS-lncRNAs, with values ranging from 1 < *R* ^2^ > 0.74 for BAALC/BAALC-AS1 (*R* ^2^ = 0.997), BAALC/BAALC-AS2 (*R* ^2^ = 0.971), HIF1A/HIF1A-AS3 (*R* ^2^ = 0.991), TNFRSF14/TNFRSF14-AS1 (*R* ^2^ = 0.956), IRF1/IRF1-AS1 (*R* ^2^ = 0.993), CA3/CA3-AS1 (*R* ^2^ = 0.976), IL1R1/IL1R1-AS1 (*R* ^2^ = 0.966), IL21R/IL21R-AS1 (*R* ^2^ = 0.98), RORA/RORA-AS1 (*R* ^2^ = 0.994), and HIF1A/HIF1A-AS1 (*R* ^2^ = 0.744) pairs.

We highlighted exclusively regulated mRNA-lncRNA pairs in *Li-*infected macrophages, such as IL21R/IL21R-AS1, HIF1A/HIF1A-AS1, HIF1A/HIF1A-AS3, and IRF1/IRF1-AS1. These results suggest that modulation of these genes may be linked to the specificity of the pathophysiology of each *Leishmania* species.

We used the NcPath’s (Li et al., 2022) database of experimentally validated and predicted lncRNA-protein coding gene interaction to investigate these lncRNA targets by mechanisms other than cis regulation. To investigate their possible function, we submitted the putative target genes to Reactome enrichment of immune response-related pathways (Table 2). We found 15 significantly over represented pathways that can be regulated by the abovementioned lncRNAs, including key recognition receptors, such as TCRs, CLRs and NLRs, and cytokine signaling by interleukins and interferon.

**TABLE 2:** Enrichment analysis of putative lncRNA targets from NcPath database.

Signaling by Interleukins
BAALC-AS1/HIF1A-AS1/HIF1A-AS3/TNFRSF14-AS1/IRF1-AS1/IL1R1-AS1/IL21R-AS1/RORA-AS1		
P(adj)	*q* value	Targets
3.28 × 10^−11^	1.81 × 10^−11^	NOD2/IL16/HIF1A/CSF1/PSMA4/ITGB1/IKBKB/CXCL8/CCL3L1/PSMC3/APP/CSF3/TYK2/STAT6/IL4R/HCK/PTPRZ1/MAPK1/SQSTM1/HSP90AA1/MEF2A/TAB1/CDC42/SOD2/TBK1/STAT1/AIP/CDKN1A/ATF2/IL15/PSMA1/PSMD14/PSMD1/PSMA3/PSMB5/UBC/GRB2/NFKB2/STAT2/PAK2/NOD1/FBXW11/CUL1/VIM/BIRC5/FLT3LG/ATF1/VAMP2/STX4/INPPL1/IL11RA/PSME1/PTPN13/PTPN2/IL1RL2/IL1R1/IL21R/PIK3R3/MAP2K1/MAP2K7/POU2F1/BCL2L1/LYN/FYN/STAT5B/IL23A/PSMB4/PSMD3/PSMD13/PSMD7/PSMD9/PSMC4/PSMD4/UBA52/RORA/SOS2/PPP2R1A/NFKBIB/JAK1/RAG1/CRKL/IRAK2/CANX/MAP3K3/RPLP0/PSME3/CSF3R/IL1RAP/SOCS5/PSME2/CAPZA1
DDX58/IFIH1-mediated induction of interferon-alpha/beta		
BAALC-AS1/TNFRSF14-AS1/IRF1-AS1/RORA-AS1		
P(adj)	q value	Targets
3.89 × 10^−08^	2.14 × 10^−08^	NLRX1/IKBKB/TRAF2/IRF3/APP/UBA7/CASP10/TNFAIP3/DHX58/TANK/IRF7/TBK1/TRAF3/UBC/NFKB2/DDX58/UBE2D3/ATG5/SIKE1/ITCH/UBA52/NFKBIB/TRIM25/TKFC/PCBP2/TAX1BP1
Interferon Signaling		
TNFRSF14-AS1/IRF1-AS1/RORA-AS1		
P(adj)	*q* value	Targets
3.98 × 10^−08^	2.19 × 10^−08^	IRF3/HLA-F/HLA-B/HLA-DRB5/TPR/UBA7/TYK2/CIITA/KPNB1/IP6K2/PLCG1/IRF7/HLA-A/HLA-DPB1/STAT1/EIF2AK2/SEC13/NUP133/NUP214/NUP205/UBC/IRF9/SP100/DDX58/PML/BST2/IFNAR1/STAT2/IRF1/MX1/ADAR/EIF4E/GBP4/GBP1/PTPN2/NUP107/NUP58/RANBP2/NUP85/NUP188/UBA52/JAK1/TRIM25/NEDD4/PIAS1/EIF4E2/PPM1B
TNFR2 non-canonical NF-kB pathway		
HIF1A-AS1/TNFRSF14-AS1/IRF1-AS1/IL21R-AS1/RORA-AS1		
P(adj)	*q* value	Targets
5.10 × 10^−08^	2.81 × 10^−08^	PSMA4/TRAF2/TNFSF11/PSMC3/TNFRSF14/CD40/TRAF3/PSMA1/PSMD14/PSMD1/PSMA3/PSMB5/UBC/NFKB2/FBXW11/CUL1/PSME1/TNFSF12/CD27/PSMB4/PSMD3/PSMD13/PSMD7/PSMD9/PSMC4/PSMD4/UBA52/BIRC2/PSME3/PSME2
Class I MHC mediated antigen processing and presentation		
HIF1A-AS1/TNFRSF14-AS1/IRF1-AS1/RORA-AS1		
P(adj)	*q* value	Targets
1.41 × 10^−07^	7.76 × 10^−08^	PSMA4/TAP2/ITGB5/IKBKB/HLA-F/HLA-B/PSMC3/UBA7/UBE3A/TAPBP/RBCK1/RNF7/ANAPC1/KEAP1/MIB2/HLA-A/PSMA1/PSMD14/PSMD1/PSMA3/PSMB5/SEC13/UBC/TAP1/SEC22B/SEC24D/UBE2D3/FBXW11/CUL1/MRC2/SEC61G/SEC61A2/CDC27/ANAPC4/ANAPC10/ANAPC5/CDC16/STX4/PSME1/ITGAV/ITCH/PSMB4/PSMD3/PSMD13/PSMD7/PSMD9/PSMC4/PSMD4/UBA52/SKP2/SEC24A/SEC24C/FBXO22/UBE2V2/SEC61B/CUL5/CANX/CBLB/NEDD4/PSME3/LNPEP/FBXL3/ATG7/VAMP8/SNAP23/LMO7/PSME2/FZR1/FBXO32
C-type lectin receptors (CLRs)		
HIF1A-AS1/TNFRSF14-AS1/IRF1-AS1/RORA-AS1		
P(adj)	q value	Targets
5.52 × 10^−06^	3.04 × 10^−06^	PSMA4/IKBKB/ITPR3/PSMC3/TAB1/PPP3R1/PSMA1/PSMD14/PSMD1/PSMA3/PSMB5/UBC/NFKB2/PAK2/FBXW11/CUL1/CLEC7A/PSME1/ITPR1/PPP3CA/LYN/FYN/ITPR2/PSMB4/PSMD3/PSMD13/PSMD7/PSMD9/PSMC4/PSMD4/UBA52/PSME3/PSME2
TCR signaling		
HIF1A-AS1/TNFRSF14-AS1/IRF1-AS1/RORA-AS1		
P(adj)	*q* value	Targets
1.82 × 10^−05^	1.00 × 10^−05^	PSMA4/IKBKB/HLA-DRB5/PSMC3/PLCG1/HLA-DPB1/PSMA1/PSMD14/PSMD1/PSMA3/PSMB5/UBC/PAK2/FBXW11/CUL1/LAT/PSME1/EVL/PTEN/PSMB4/PSMD3/PSMD13/PSMD7/PSMD9/PSMC4/PSMD4/UBA52/PSME3/PSME2
Signaling by the B Cell Receptor (BCR)		
HIF1A-AS1/TNFRSF14-AS1/IRF1-AS1/RORA-AS1		
P(adj)	q value	Targets
6.08 × 10^−05^	3.34 × 10^−05^	PSMA4/IKBKB/ITPR3/PSMC3/NFKBIE/RASGRP3/PPP3R1/PSMA1/PSMD14/PSMD1/PSMA3/PSMB5/UBC/GRB2/FBXW11/CUL1/STIM1/PSME1/ITPR1/PPP3CA/LYN/FYN/ITPR2/PSMB4/PSMD3/PSMD13/PSMD7/PSMD9/PSMC4/PSMD4/UBA52/NFKBIB/PSME3/PSME2/SH3KBP1
MHC class II antigen presentation		
TNFRSF14-AS1/IRF1-AS1/RORA-AS1		
P(adj)	*q* value	Targets
2.36 × 10^−04^	1.30 × 10^−04^	HLA-DMA/HLA-DRB5/HLA-DMB/KLC4/ACTR1B/CD74/KIF3A/HLA-DPB1/KLC1/SEC13/DCTN1/AP2B1/CLTA/RAB7A/SEC24D/AP1M1/CTSB/DCTN5/CLTC/SEC24A/SEC24C/CANX/CAPZA1/CAPZA2
Fc epsilon receptor (FCERI) signaling		
HIF1A-AS1/TNFRSF14-AS1/IRF1-AS1/RORA-AS1		
P(adj)	*q* value	Targets
9.57 × 10^−04^	5.26 × 10^−04^	PSMA4/IKBKB/ITPR3/PSMC3/MAPK1/TAB1/PPP3R1/PLCG1/PSMA1/PSMD14/PSMD1/PSMA3/PSMB5/UBC/GRB2/PAK2/FBXW11/CUL1/LAT/PSME1/MAP2K7/ITPR1/PPP3CA/LYN/FYN/ITPR2/PSMB4/PSMD3/PSMD13/PSMD7/PSMD9/PSMC4/PSMD4/UBA52/PSME3/PSME2
Toll-like Receptor Cascades		
BAALC-AS1/TNFRSF14-AS1/IRF1-AS1/RORA-AS1		
P(adj)	*q* value	Targets
3.17 × 10^−03^	1.74 × 10^−03^	NOD2/IKBKB/IRF3/APP/MAPK1/MEF2A/TAB1/TANK/IRF7/TBK1/TRAF3/ATF2/UBC/NFKB2/NOD1/UBE2D3/FBXW11/CUL1/ATF1/CTSB/MAP2K1/MAP2K7/UBA52/PPP2R1A/NFKBIB/BIRC2/IRAK2
Costimulation by the CD28 family		
TNFRSF14-AS1/IRF1-AS1/RORA-AS1		
P(adj)	*q* value	Targets
5.27 × 10^−03^	2.90 × 10^−03^	HLA-DRB5/TNFRSF14/PDCD1/PRR5/CDC42/HLA-DPB1/GRB2/PAK2/PIK3R3/AKT3/LYN/FYN/TRIB3/PPP2R1A/PPP2R5A/PPP2R5B
Nucleotide-binding domain, leucine rich repeat containing receptor (NLR) signaling pathways		
BAALC-AS1/TNFRSF14-AS1/IRF1-AS1/RORA-AS1		
P(adj)	q value	Targets
1.05 × 10^−02^	5.79 × 10^−03^	NOD2/IKBKB/APP/TNFAIP3/TAB1/NFKB2/NOD1/BCL2L1/ITCH/BIRC2/IRAK2/SUGT1
Fcgamma receptor (FCGR) dependent phagocytosis		
TNFRSF14-AS1/IRF1-AS1/CA3-AS1/RORA-AS1		
P(adj)	*q* value	Targets
3.32 × 10^−02^	1.83 × 10^−02^	ITPR3/HCK/PLD1/PLA2G6/MAPK1/HSP90AA1/CDC42/PLCG1/GRB2/MYO1C/ELMO2/PTK2/ITPR1/LYN/ABL1/FYN/ITPR2/ACTR3/MYO10/ACTR2/MYO5A/BRK1/WASF1/ARPC1A/LIMK1
Growth hormone receptor signaling		
BAALC-AS1/TNFRSF14-AS1/IRF1-AS1/RORA-AS1		
P(adj)	*q* value	Targets
4.99 × 10^−02^	2.74 × 10^−02^	PRLR/SH2B1/MAPK1/STAT1/LYN/STAT5B

## 3 Discussion

LncRNAs interfere with gene expression at multiple levels and affect the transcriptome by controlling transcription and mRNA stability in *cis* in *trans* (Fernandes et al., 2019). To see if lncRNAs may play a role in *Leishmania*-elicited macrophage responses, we analyzed the profile of host mRNAs and lncRNAs. For the first time, we showed species-specific regulation of host RNA expression in human macrophages infected with *L. amazonensis*, *L. braziliensis,* or *L. infantum*. Interestingly, even though *La* and *Lb* belong to different subgenera, the gene expression pattern of macrophages infected with those species are more similar than *Li*, indicating that gene expression can reflect more the different pathologies rather than evolutionary proximity.

We used total RNA depleted of rRNA for RNA-seq to address the analysis of both polyadenylated and non-polyadenylated RNAs, including lncRNAs from excised introns or some non-polyadenylated intergenic lncRNAs (Pinkney et al., 2020; L. Yang et al., 2011), facilitating a broader analysis of lncRNA expression in *Leishmania-*infected macrophages. With this, our transcriptome mapped 24% of total identified DE transcripts to lncRNAs, being the second most abundant class of RNA in our data after rRNA depletion (Table 1), revealing that a significant fraction of the transcriptome was unappreciated in previous studies from this field (Salloum et al., 2021). LncRNAs were also investigated in the genome of *Lb* (Ruy et al., 2019), *L. major,* and *L. donovani* (Freitas Castro et al., 2017). Together, these recent studies open a field to study the interplay of host-parasite ncRNAs.

Regarding our dataset, it is important to emphasize that there was no enrichment for small RNAs for library construction, as required for the proper identification of mature miRNAs, so the fraction of these molecules shown in Table 1 must be interpreted with caution. The literature already established that miRNAs are essential for macrophage reprogramming during *Leishmania* infection (Paul et al., 2020). Moreover, as a new class of DE transcripts, we observed regulation of lncRNAs that are host genes for miRNAs (Supplementary Table S1) displaying important functions in inflammation as recently described in the literature. Our data show an upregulation of the well know inflammation-related miR-155 (Supplementary Table S1) as well as its host gene MIR155HG, which is involved in pro-inflammatory response during chronic obstructive pulmonary disease (Li et al., 2019) and against influenza A virus (Maarouf et al., 2019). Recent work shows MIR155HG as a suppressor of dendritic cell-mediated autoinflammation (Niu et al., 2020). Also, MIR210HG was upregulated in our infection models and was described to act together with HIF1α to promote glycolysis during cancer, revealing that lncRNAs may be involved in regulating metabolic pathways (Du et al., 2020).

To further understand the distinct trends in mRNA and lncRNA regulation, we compared the host gene expression in the infection by different *Leishmania* species. With this, we identified commonly induced lncRNAs (Figures 1A,B) that may control pathways mechanistically shared by distinct species to circumvent the immune response. On the other hand, the unique sets of lncRNAs regulated in *Li*-infected macrophages or shared between *La* and *Lb* only could unravel regulatory modules related to specificities of clinical manifestations. Although the total number of DE mRNAs and lncRNAs is similar among *La*, *Lb,* and *Li*-infected macrophages (Supplementary Table S1), we observed a higher fraction of those specific for *Li* (Figures 1A,B). The difference is also evident in the multivariate analysis by PCA (Supplementary Figure S2), suggesting that the separation of clusters from *La* and *Lb* infection from *Li* infection reflects the host’s gene expression modulation specificities, leading to distinct physiopathological outcomes caused by these *Leishmania* species.

Since lncRNA classification can contribute to interpreting its function (Fernandes et al., 2019), we showed that most of these transcripts are intergenic or antisense to protein-coding genes (Figure 1C). The high proportion of these types of lncRNAs is following the observed in the whole blood transcriptome, mainly composed of neutrophils and T cells, in visceral leishmaniasis patients infected with *L. infantum* (Maruyama et al., 2022). The prevalence of AS lncRNAs is in accordance with a study showing that over 20% of human transcripts pair to AS gene expression (Chen et al., 2004).

We first compared macrophage phenotype upon infection with *La*, *Lb,* and *Li* based on the protein coding subset of genes. Our analysis showed that *Li* infection elicits a response with higher NES scores than *La* and *Lb* infection for immune response-related Reactome pathways (Figure 2). We also depicted results in Alluvial plots facilitating further discussion into specificities of DEGs among infection by different *Leishmania* species (Figure 3), since to our knowledge this is the first study to compare the transcriptome of *La*, *Lb* and *Li*-infected macrophages. For this, we included pathways from the cellular response to stimuli from Reactome. Many of the identified DEGs were already identified in multiple models of *Leishmania* infection.

Here, we identified upregulation NCF1 (Figures 3A,B), corresponding to the gp47 subunit of the NADPH oxidase (NOX), that was previously shown as essential for ROS production in murine neutrophils upon *La* infection (Carlsen et al., 2013). The NOX components NCF1, NCF2 (gp67), and CYBB (gp91) also lead to ROS production against *Lb* in human monocytes activated by IFN-γ and in cutaneous lesions (Novais et al., 2014). We also found other markers induced in the transcriptome, such as TNF-α, STAT1, and STAT4 (Novais et al., 2014). On the other hand, we identified enrichment of the detoxification of ROS pathways (Figure 3B). In the literature, the antioxidant response to *La* infection was evaluated at a systemic level, showing increased SOD2 levels and activity in the liver of infected mice (Gasparotto et al., 2017). Our data also corroborates data from *L. major*-infected BALB/c or C57BL/6 macrophages, where GSR was upregulated (Bichiou et al., 2021). The increased level of detoxification molecules agrees with high glutathione levels in the *La*-infected macrophages (Muxel et al., 2019; Mamani-Huanca et al., 2021).

There was an exclusive regulation of mRNAs related to the cellular response to hypoxia, such as UBC, HIF1A, EPAS1/HIF2A, and the proteasome-related PSME1, PSME2, PSMA6 by *Li*-infected macrophages (Figure 3C). In previous studies on visceral leishmaniasis models, HIF1α was essential for a host-protective response during *L. donovani* infection *in vitro* and *in vivo* (Mesquita et al., 2020). However, during *L. major* infection, *Hif1a* mRNA was only observed upon LPS + IFN-γ treatment or hypoxia condition (Schatz et al., 2016), probably because some *Leishmania* species can interfere in its stabilization. HIF1α binds to hypoxia response elements (HRE)-containing target genes, regulating the transcription of genes glucose transporter 1 (GLUT1), hexokinase II, pyruvate dehydrogenase kinase 1 and lactate dehydrogenase A, and glycolysis itself (Wheaton & Chandel, 2011), controlling pro-inflammatory response of macrophages (Tannahill et al., 2013).

The TNF and TNFR2 signaling are also prevalent pathways during *Li* infection. Previously published transcriptome of *Li*-infected THP-1 macrophages showed TNFAIP3 and IRF7 upregulation but not IL1B transcript, as we found (Supplementary Table S1). The difference can be due to different procedures and the *Leishmania* strain used for the experiments (Gatto et al., 2020).

To understand the implication of coexpressed protein coding mRNAs and closely located lncRNAs (Figure 4D), we ran a coexpression analysis showing that the fold change of these mRNA-lncRNA pairs is positively-correlated (Figures 4A–C). This approach led us to spot interesting correlations and to find the reported lncRNAs in previously published data.

Manual inspection of the available DEG table from the previously published transcriptome (Fernandes et al., 2016) allowed the identification of four antisense lncRNAs regulated after 24 h of *La* infection in primary human macrophages: the upregulation of BAALC antisense RNA 2 (BAALC-AS2) and GSN antisense RNA 1 (GSN-AS1) and downregulation of BAIAP2 antisense RNA 1 (BAIAP2-AS1) and the solute carrier family 22 member 18 antisense (SLC22A18AS) (Fernandes et al., 2016). Also, they found upregulation of both protein-coding BAALC and BAALC-AS2 after 4 and 24 h of *La* and *L. mexicana* infection. In our study, BAALC/BAALC-AS1 and BAALC/BAALC-AS2 pairs were upregulated in *La*, *Lb,* and *Li* infection. BAALC is a binder of MAP3K1 and KLF4, previously described in cancer as interacting with these molecules and inhibiting their functions (Morita et al., 2015). MAP3K1 is involved in MAPK signaling in response to pro-inflammatory stimuli (Otto et al., 2021). It can be directly targeted by miR-770, reducing the M2 polarization of macrophages (Liu et al., 2021). During *Leishmania* infection, MAP3K1 appears to have species-specific expression, since it is upregulated by *L. mexicana* and downregulated by *L. donovani* and could be involved in type I interferon production in dendritic cells (Favila et al., 2014). During *La* infection, MAPK activation is required for IL-10 production (Yang et al., 2007). The other target, KLF4, is essential for the M2 polarization of macrophages (Lin et al., 2021).

We saw downregulation of the RORA/RORA-AS1 pair in *La* and *Li*, but not in *Lb*-infected macrophages. The RORα is a transcription factor that negatively regulates inflammation, and its knockout in THP-1 significantly increases TNF, IL1β, and IL-6 production upon LPS stimulation (Nejati Moharrami et al., 2018). The RORα function in promoting M2 macrophage polarization is related to KLF4 (Han et al., 2017), the abovementioned BAALC target. However, no study has explored the function of RORA-AS1 yet.

Maruyama *et al* also found CA3-AS1 and IRF1-AS1 downregulated in the serum of patients with active visceral disease caused by *Li versus* controls (Maruyama et al., 2022). While the CA1/CA3-AS1 pair was upregulated in the blood of visceral leishmaniasis patients (Maruyama et al., 2022), in our study, the CA3/CA3-AS1 pair is downregulated by *La*, *Lb,* and *Li* infection. The CA3-AS1 was shown to act as a sponge of the miR-93 (Zhang et al., 2020), while CA3 is a known antioxidant in different cell types (di Fiore et al., 2018), however, both transcripts lack functional studies in macrophages. The IRF1/IRF1-AS1 pair appear upregulated exclusively in *Li*-infected macrophages in our coexpression analysis (Figures 4A–C). IRF1-AS1 is induced by IFNα and was shown to be essential for its signaling and NF-κB-mediated response, mediating the transcription of the IRF1 gene (Barriocanal et al., 2022), as shown to be correlated in our results. The upregulation of IRF1 prevents immunopathology in *Li*-infected mice (Sacramento et al., 2020).

Interestingly, the HIF1A, HIF1A-AS1 and HIF1A-AS3 transcripts were upregulated only in *Li*-infected macrophages. HIF1A-AS1 remains with unknown functions. In cancer cells, the HIF1A-AS3 is induced and stabilizes HIF1A binding in HRE by HIF1A-AS3 assembling the HIF1A transactivation complex and enhancing HIF1α target genes (Zheng et al., 2021).

We also highlighted cytokine receptors in our coexpression analysis (Figure 4). Upregulation of the IL1R1 occurred in all three *Leishmania* infection models. However, other species, like *L. donovani* can impair IL1R1 activity at multiple levels to survive in macrophages (Parmar et al., 2018). We also could not find publications on the IL1R1-AS1 function. On the IL-2 cytokine family receptors, IL21R/IL21R-AS1 pair was downregulated only in *Li*-infected macrophages. The IL21R is dispensable in the cutaneous leishmaniasis model by *L. major* (Fröhlich et al., 2007). Also, different from our finding, a previous study found an inverse expression level of the IL21R/IL21R-AS1 (Riege et al., 2017). The study of Maruyama *et al* also found modulation of the antisense to the cytokine IL21, the IL21-AS1 lncRNA (Maruyama et al., 2022). The upregulation of TNFRSF14/TNFRSF14-AS1 pair occurred upon *Lb* and *Li* infection. An independent study showed the upregulation of TNFRSF14 with *Li*-infected THP-1 (Gatto et al., 2020). The TNFRSF14-AS1 is poorly studied, with one report indicating it as a prognostic marker in breast cancer (Dashti et al., 2020; Lv et al., 2021).

Interestingly, we found that 13 of 15 pathways targeted by the highlighted AS-lncRNAs (Table 2) are also enriched in the analysis of protein coding genes (Figure 2). Both TNFRSF14-AS1 and IRF1-AS1 are upregulated in *Li*-infected macrophages and are involved in MHC-II antigen presentation. However, this pathway is only enriched during *La*- and *Lb*-infected macrophages in our analysis of protein-coding genes, indicating that a regulation with negative effects can be investigated for those targets. Some of the putative target genes related to the TNFR2 non-canonical NF-kB pathway, such as the UBC, are depicted in the alluvial plots as regulated at transcriptional level (Figure 3), however, since lncRNA have multiple mechanisms, we cannot exclude regulation at protein level or activity. Finally, this analysis attributed functions related to immune response to the lncRNAs CA3-AS1 and BAALC-AS1, that were previously shown in the transcriptome of *Leishmania*-infected macrophages.

We show that the lncRNA signature is altered during macrophage infection with *Leishmania* in a species-specific pattern. We could also detect coexpressed pairs of protein-coding mRNAs and proximal AS lncRNAs suggesting cis regulatory roles in the host-parasite interface. We also included pathway analysis of lncRNA putative targets indicating association with genes of the immune response at multiple regulatory levels. However, further validations and experiments are necessary to unravel regulations, molecular mechanisms, and implications of lncRNAs in the response of macrophages to *Leishmania* infection.

## 4 Materials and methods

### 4.1 Cell culture and infection

Five samples were prepared for each group (uninfected, *La*-infected, *Lb*-infected, and *Li*-infected) for RNA-seq performance. From the THP-1 monocytic cell line (TIB-202-ATCC, ATCC, United States), 5×10^6^ cells were counted in a Neubauer chamber and plated in 6-well plates (SPL Life Sciences, Korea). Monocytes were then differentiated in macrophage-like cells with 30 ng/ml PMA (Sigma Aldrich, St. Louis, MO) in RPMI 1640 (Gibco, Grand Island NY, United States) supplemented with heat-inactivated 10% fetal bovine serum (FBS) (Gibco, São Paulo, BR) for 72 h following 72 h resting with PMA-free media at 34°C/5%CO_2_.

*Leishmania* promastigote forms were cultivated in M199 medium (Gibco, Grand Island NY, United States), pH 7.0, supplemented with L-glutamine, 10% heat-inactivated FBS, 0.25% hemin, 40 mM NaHCO3, 100 μM adenine, 40 mM HEPES, 100 U/ml penicillin, and 100 μg/ml streptomycin (Gibco) at 25°C and split weekly.

Differentiated THP-1 were infected (MOI 5:1) with parasites in the stationary phase (day 7) from either *Leishmania amazonensis* (MHOM/BR/1973/M2269), *Leishmania braziliensis* (MHOM/BR/1975/M2903), or *Leishmania infantum* (MCER/BR/1981/M6445) species. After 4 h, extracellular parasites were washed with PBS 1X, and cells were maintained in fresh media for 20 h.

### 4.2 Intracellular parasites count

For infection count, parasites were marked with the Vybrant CFDA SE Cell Tracer Kit (Invitrogen) with the proportion of 1×10^8^ parasites/mL/5 μM CFDA SE reagent for 20 min at 25°C. After, cells were washed with both FBS-supplemented RPMI and PBS1X before infection. About 1×10^6^ THP-1-derived macrophages were infected with CFDA SE-labeled *Leishmania* in 24-well plates. Cells were harvested after 24 h of infection using PBS/EDTA 1 mM treatment for detachment and 4% PBS/paraformaldehyde (PFA) for fixation. Cells were resuspended in 25μL of PBS 1X, and images were obtained in the FlowSight® imaging cytometer. Data analysis was performed in the IDEAS software using Wizard Spot Count. The number of intracellular parasites per cell ranged from 2.7 to 4.2, and the percentage of infected macrophages ranged from 26 to 56 (Supplementary Figure S1).

### 4.3 RNA extraction and RNA-seq

Total RNA extraction from five independent biological replicates of each infected and uninfected macrophage was performed 24 h after infection using TRIzol reagent (Life Technologies, Carlsbad, CA, United States) following manufacturer instructions.

After, RNA samples were treated with DNase I (1 U/µg of RNA) (Thermo Scientific, Lithuania, EU) at 37°C for 1 h. The absence of DNA contamination was determined from the A260/A280 ratio using a spectrophotometer Nanodrop ND1000 (Thermo Scientific, United States). RNA integrity was evaluated using an Agilent 2,100 Bioanalyzer and a Pico Agilent RNA 6000 kit (Agilent Technologies, Santa Clara, CA, United States). rRNA depletion was performed using Ribo-Zero Human/rat/mice Ribo-Zero plus rRNA Depletion Kit (Illumina). Library preparation was performed using 1 µg of rRNA-depleted total RNA using Truseq Stranded Total RNA LT Sample Prep Gold kit, without molecular barcodes. Sample preparation followed preparation using the NovaSeq 6000 S4 Reagent Kit (Illumina) according to manufacturer’s instructions for RNA sequencing submission. The sequencing was performed with paired-ends (100 bp) using the Illumina Novaseq 6,000 Platform in Macrogen Inc. Service (Seoul, South Korea).

#### 4.3.1 RNA-seq analysis

Quality control of raw sequencing data was done using FastQC tool. Mapping to a human reference genome assembly (GRCh38) was done using bowtie2 (Langmead & Salzberg, 2012). Read counts from the resulting BAM alignment files were obtained with featureCounts using a GTF gene annotation from the Ensembl database (Yang et al., 2014; Howe et al., 2021). The R/Bioconductor package edgeR was used to identify differentially expressed genes among the samples after removing absent features (0 counts) (McCarthy et al., 2012). Genes with adjusted *p* values less than 0.05 were identified as differentially expressed. For each *Leishmania*-infected group comparison, gene set enrichment analysis was performed using the fgsea R package. Genes with Ensembl IDs were transformed into gene symbols by the biomaRt package (Steffen et al., 2009) and ordered by their log FC values. The identified lncRNAs were classified using the GRCh38.p14 database source table with data for lncRNA annotation according to the genomic position. These annotations as intergenic, antisense, overlapping, intronic, and non-classified, and the transcripts that lack validation studies were referred to as novel transcripts. For the enrichment analysis of protein-coding transcripts, pre-ranked genes and Reactome gene sets from Enrichr (Chen et al., 2013) were provided to GSEA (Subramanian et al., 2005), with remaining default parameters. To identify significant common pathways between all comparisons, pathways with a *p*-value below a threshold of 0.05 for at least 1 comparison were selected and clustered based on the NES with hierarchical clustering. Correlation plots were generated to display the NES values using the corrplot package. The molecular degree of perturbation for each was assessed for each *Leishmania*-infected group samples relative to the uninfected control group samples using mdp R package (Gonçalves et al., 2019). The Pearson correlation test (|R| > 0.8) was used to identify the association between 10 pairs of lncRNAs (BAALC-AS1, BAALC-AS2, HIF1A-AS1, HIF1A-AS3, TNFRSF14-AS1, IRF1-AS1, IL1R1-AS1, IL21R-AS1, RORA-AS1, and CA3-AS1) and mRNAs (BAALC, HIF1A, TNFRSF14, IRF1, IL1R1, IL21R, RORA, and CA3). These lncRNAs’ predicted targets were retrieved from the NcPath database (Li et al., 2022) and submitted to GSEA based on Reactome gene sets for enrichment analysis.

## Data Availability

The high-quality reads were deposited at NCBI under BioProject (https://www.ncbi.nlm.nih.gov/) with the accession number PRJNA881925.

## Supplementary material

Supplementary Figure S1 Infectivity of *Leishmania amazonensis (La)*, *Leishmania braziliensis (Lb)* and *Leishmania infantum (Li)* in THP-1 derived macrophages. **(A)** percentage of infected macrophages and **(B)** amastigotes per macrophage (*N* = 6 each).

Supplementary Figure S2 RNA-seq Quality Control. **(A)** Boxplots for molecular degree of perturbation (MDP) scores and **(B)** PCA for *Leishmania amazonensis (La), Leishmania braziliensis (Lb) and Leishmania infantum (Li)*-infected THP-1 derived macrophages (*N* = 5 each).

Supplementary Table S1 Differentially expressed (DE) genes from *La*-, *Lb*- and *Li*-infected compared to non-infected THP-1 macrophages.

Supplementary Table S2 Gene set enrichment analysis (GSEA) for THP-1 macrophages infected with *La, Lb and Li*.
