# Supplementary figures and images for: Comparative transcriptomic analysis of long noncoding RNAs in Leishmania-infected human macrophages
Source: Front Genet. 2023 Jan 4;13:1051568. doi: 10.3389/fgene.2022.1051568 (PMC9845402; doi:10.3389/fgene.2022.1051568)

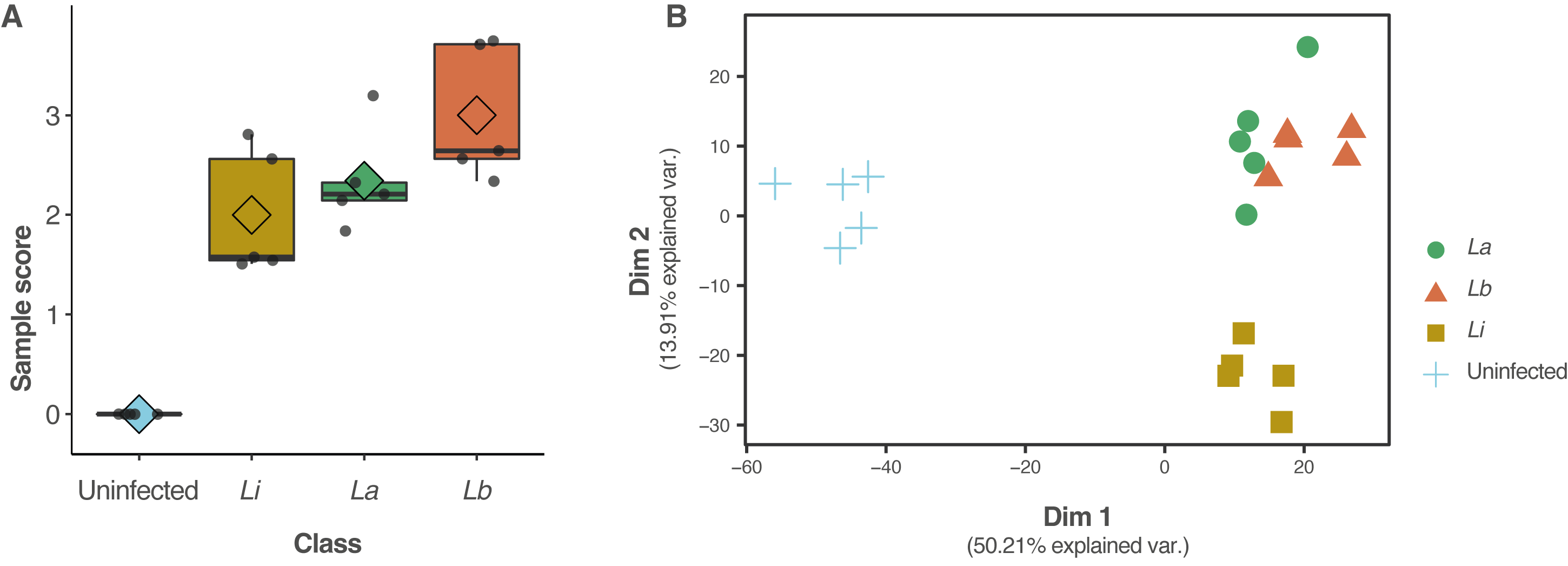

Supplement: Supplementary file 2 [file Image2.tif]

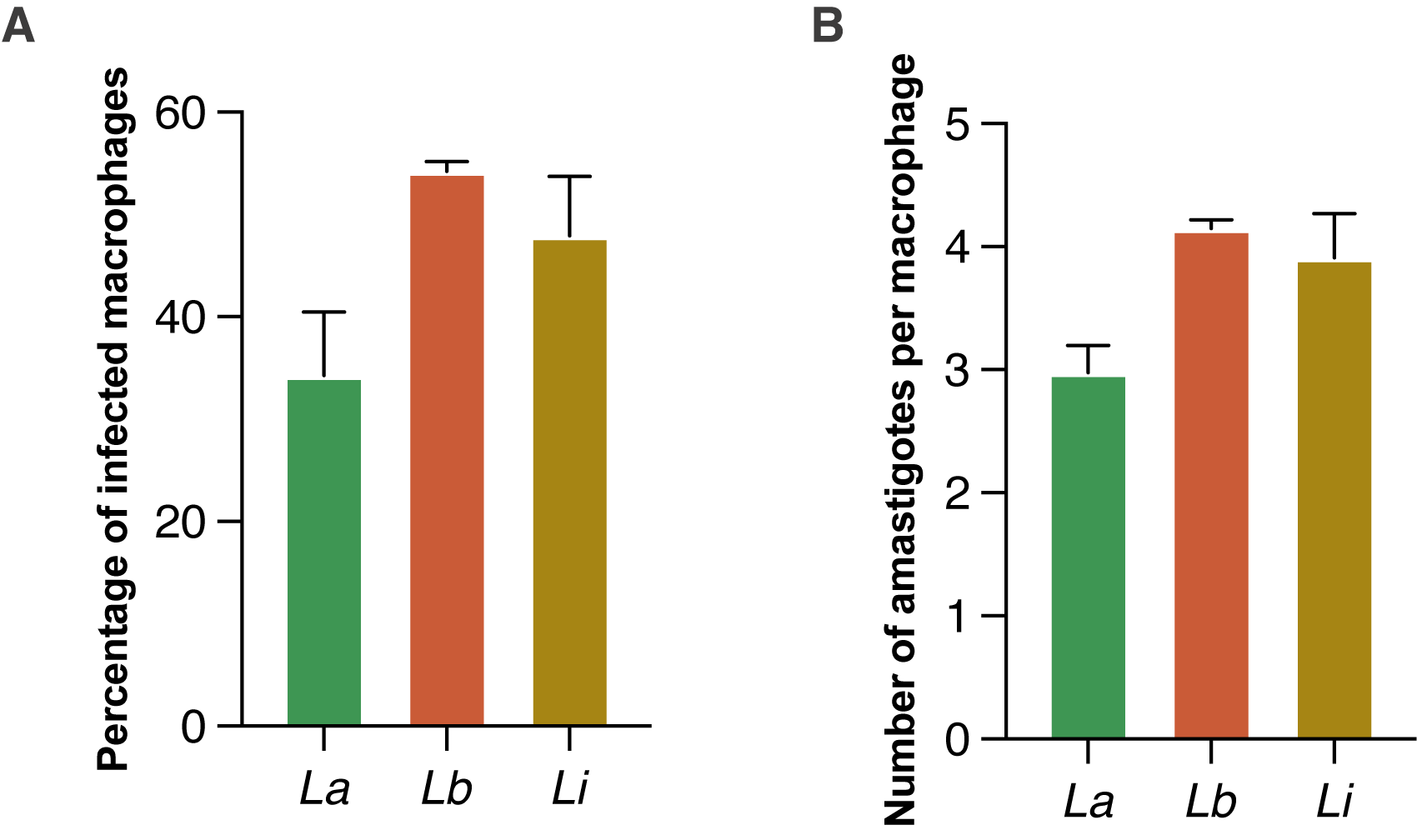

Supplement: Supplementary file 3 [file Image1.tif]
